# Esophagectomy performed at institutes certified by the Japan Esophageal Society provide long-term survival advantages to esophageal cancer patients: second report analyzing 4897 cases with propensity score matching

**DOI:** 10.1007/s10388-019-00712-w

**Published:** 2020-01-14

**Authors:** Satoru Motoyama, Eri Maeda, Masahiko Yano, Takushi Yasuda, Masaichi Ohira, Yoshiaki Kajiyama, Takahiro Higashi, Yuichiro Doki, Hisahiro Matsubara

**Affiliations:** 1 The Japan Esophageal Society, Tokyo, Japan; 2 Department of Environmental Health Science and Public Health, Akita University Graduate School of Medicine, Akita, Japan; 3 Division of Health Services Research, Center for Cancer Control and Information Services, National Cancer Center, Tokyo, Japan; 4 Esophageal Surgery, Akita University Graduate School of Medicine, 1-1-1 Hondo, Akita, 010-8543 Japan

**Keywords:** Esophageal cancer, Esophagectomy, Institute, Certification, Survival, Propensity score matching

## Abstract

**Background:**

It will be important for the Japan Esophageal Society (JES) to show an evident advantage of its institution certification system. To achieve this essential task, we used nationally acquired big data to re-analyze 5-year survival information.

**Methods:**

In 2008–2009, there were 4897 thoracic esophageal cancer patients who underwent esophagectomy and were registered in the National Database of Hospital-based Cancer Registries. We divided these patients into two groups, those who underwent surgery at an Authorized Institute for Board Certified Esophageal Surgeons (AIBCES) or a Non-AIBCES. We then compared the patient backgrounds and 5-year survival rates between these two groups, with and without propensity score matching.

**Results:**

There were 3080 (63%) patients who underwent esophagectomy at an AIBCES and 1817 (37%) who underwent surgery at a Non-AIBCES. Comparison of the Kaplan–Meier survival curves using log-rank tests indicated a significant difference between the AIBCES and Non-AIBCES groups at all cStages (cStages I–IV). Multivariable Cox proportional hazard analysis stratified by clinical stage and adjuvant treatment revealed that AIBCES vs. Non-AIBCES is a significant independent factor (adjusted HR 0.78) for survival. After propensity score matching ensuring the backgrounds of the two groups being equivalent, there were significant differences in the 5-year survival rates for patients with cStages I–III disease between the AIBCES and Non-AIBCES groups.

**Conclusions:**

There is a survival advantage to undergoing esophagectomy at an AIBCES. The institute certification system from the JES will contribute to the future establishment of a more appropriate surgery delivery system for thoracic esophageal cancer.

## Introduction

The Japan Esophageal Society (JES) recently reported the appropriateness of the institute certification system for esophageal surgeries established by the society itself [1]. Using data from the National Database of Hospital-based Cancer Registries 2008 along with 5-year survival information, it was demonstrated that the 5-year survival rate among cStage II–III thoracic esophageal cancer patients treated with esophagectomy at an “Authorized Institute for Board Certified Esophageal Surgeons (AIBCES)” was significantly better than the survival rate among patients treated at a Non-AIBCES. This important result may provide direction for building a more appropriate surgery delivery system for thoracic esophageal cancer in the future, not only for Japan but also for the world. However, this study had several limitations. First, the number of patients analyzed was somewhat small, given that the data were drawn from a large national database, and the data obtained were registered during only a single year. As a result, we did not see the significant 5-year survival difference in cStage I patients in the prior study, where most patients were treated with surgery alone. Furthermore, there was a difference between the backgrounds of patients treated at AIBCESs and Non-AIBCESs, which was especially evident in cStage II–III patients—that is, the proportion of patients who received adjuvant chemotherapy was much higher at AIBCESs than at Non-AIBCESs. It is incumbent of the JES to show an evident advantage of the institution certification system to send a strong message to Japanese society. To achieve this important task, in the present study we analyzed data from total of 4897 patients collected by the National Database of Hospital-based Cancer Registries in 2008–2009 and matched the patients’ backgrounds using a propensity score.

## Patients and methods

### National Database of Hospital-based Cancer Registries

We retrieved the 2008–2009 data from the National Database of Hospital-based Cancer Registries from the National Cancer Center, Tokyo, Japan [2]. The registry data included the following information on individual cancer patients: (i) clinical profiles, including birth date, sex, tumor topology, and histology code defined by the International Classification of Disease for Oncology, third edition (ICD-O-3); (ii) clinical and pathological tumor–node–metastasis (TNM 6th Edition) stage based on the Union for International Cancer Control (UICC) guidelines; (iii) diagnosis date; (iv) first-line treatment details; and (v) survival information (follow-up time after diagnosis of cancer). We extracted the data for patients diagnosed with thoracic esophageal cancer (C151, C153–155) and treated surgically at a registered hospital for thoracic esophageal cancer, and for patients diagnosed at another hospital but treated surgically at a registered hospital for thoracic esophageal cancer. To ensure survival data quality, the survival data analyzed were limited to that from facilities able to provide 5-year survival data for all cancers for more than 90% of their patients.

### Authorized Institute for Board-Certified Esophageal Surgeon

The JES began certifying AIBCESs in 2013. The provisions for certification are described in our previous report [1]. The first selection of AIBCESs was deliberated in 2012 and delivered in 2013. This means that AIBCESs certified in 2013 were selected based on research and clinical achievements during the period from 2007 to 2011. In the present study, the data were from AIBCES certified in 2013 or 2014 based on research and clinical achievements during 2007–2011 or 2008–2012. Note that both included data from patients treated in 2008–2009.

### Statistical analysis

We divided the patients into two groups, those who underwent surgery at an AIBCES and those who received surgery at a Non-AIBCES, and compared the patient backgrounds and 5-year survival rates between these two groups. Statistical comparisons between patients in the AIBCES and Non-AIBCES groups were carried out using Student’s *t* test, the Chi-squared test, Fisher’s exact test, or the Cuzick's Wilcoxon-type test for trend, depending on the type and distribution of the variables. Overall survival was characterized using Kaplan–Meier curves. Survival curves were compared between the two groups using the log-rank test. A multivariable Cox proportional hazards regression model was developed to evaluate the effect of treatment at an AIBCES on survival after adjusting the analysis stratified by clinical stage and adjuvant treatment. Propensity scores were calculated by fitting a multivariable logistic regression model adjusted for age, sex, clinical stage (cStage), tumor depth (cT), node status (cN), and metastasis status (cM). We conducted nearest neighbor matching within a caliper (0.01) after excluding patients whose clinical stage was unknown (*n* = 110). After checking the balance of the covariates between the two groups by comparing standardized difference, we conducted Cox proportional hazards regression analyses, stratified by adjuvant treatment [3]. A two-sided *p* value of < 0.05 was used to define statistical significance. We performed all statistical operations using STATA 14-MP (Stata Corp LP, College Station, TX, USA).

## Results

The Hospital-based Cancer Registry for 2008 and 2009 listed 4897 patients with esophageal cancer and underwent esophagectomy for thoracic esophageal cancer. There were 3080 (63%) patients who underwent surgery at an AIBCES and 1817 (37%) who underwent surgery at a Non-AIBCES. The ratio of participants treated esophagectomy at AIBCES and Non-AIBCES was not different compare to that of using the Japanese NCD between 2015 and 2017 (67% and 33%), which corresponds to > 95% of surgeries in Japan [4]. Table 1 shows the backgrounds of the patients in the two groups. There were significant differences in age, cN, cM, cStage, type of surgery and type of adjuvant therapy in the crude data.

**Table 1:** Characteristics of patients in the AIBCES or Non-AIBCES groups before and after propensity score matching

	Before					After				
	AIBCES	Non-AIBCES	Standardized difference	*P*		AIBCES	Non-AIBCES	Standardized difference	*P*	
Numbers of patients	3080	1817				1727	1727			
Factors before the assignment										
Age (mean, SD)	65.0 (8.1)	65.9 (8.3)	− 0.120	< 0.001	1)	66.2 (8.1)	65.7 (8.3)	0.062	0.070	1)
Numbers of males (%)	2618 (85.0%)	1545 (85.0%)	− 0.001	0.977	2)	1440 (83.4%)	1470 (85.1%)	− 0.048	0.161	2)
T classification				0.504	3)				0.157	3)
Tis	11 (0.4%)	5 (0.3%)	0.015			2 (0.1%)	1 (0.1%)	0.020		
T1	995 (32.3%)	515 (28.3%)	0.086			521 (30.2%)	511 (29.6%)	0.013		
T2	579 (18.8%)	469 (25.8%)	− 0.169			510 (29.5%)	453 (26.2%)	0.074		
T3	1319 (42.8%)	667 (36.7%)	0.125			596 (34.5%)	665 (38.5%)	− 0.083		
T4	121 (3.9%)	104 (5.7%)	− 0.084			96 (5.6%)	94 (5.4%)	0.005		
Tx	55 (1.8%)	57 (3.1%)	− 0.087			2 (0.1%)	3 (0.2%)	− 0.015		
N classification				0.005	3)				0.094	3)
N0	1362 (44.2%)	878 (48.3%)	− 0.082			907 (52.5%)	846 (49.0%)	0.071		
N1	1628 (52.9%)	877 (48.3%)	0.092			790 (45.7%)	859 (49.7%)	− 0.080		
N2	31 (1.0%)	18 (1.0%)	0.002			24 (1.4%)	17 (1.0%)	0.037		
N3	3 (0.1%)	2 (0.1%)	− 0.004			2 (0.1%)	1 (0.1%)	0.020		
Nx	56 (1.8%)	42 (2.3%)	− 0.035			4 (0.2%)	4 (0.2%)	0		
M classification				0.017	2)				0.973	4)
M0	2740 (89.0%)	1662 (91.5%)	− 0.085			1610 (93.2%)	1608 (93.1%)	0.005		
M1	283 (9.2%)	127 (7.0%)	0.081			116 (6.7%)	118 (6.8%)	− 0.005		
Mx	57 (1.9%)	28 (1.5%)	0.024			1 (0.1%)	1 (0.1%)	0		
Clinical stage (UICC)				0.030	3)				0.068	3)
Stage 0	12 (0.4%)	5 (0.3%)	0.020			2 (0.1%)	1 (0.1%)	0.020		
Stage I	758 (24.6%)	414 (22.8%)	0.043			433 (25.1%)	410 (23.7%)	0.031		
Stage II	1002 (32.5%)	721 (39.7%)	− 0.149			748 (43.3%)	705 (40.8%)	0.050		
Stage III	962 (31.2%)	494 (27.2%)	0.089			424 (24.6%)	489 (28.3%)	− 0.085		
Stage IV	287 (9.3%)	132 (7.3%)	0.075			120 (7.0%)	122 (7.1%)	− 0.004		
Unknown	59 (1.9%)	51 (2.8%)	− 0.059			-	-			
Factors after the assignment										
Surgery				0.002	2)				0.033	4)
Thoracotomy	2467 (80.1%)	1522 (83.8%)	0.094			1390 (80.5%)	1440 (83.4%)	− 0.076		
Thoracoscopic	602 (19.5%)	285 (15.7%)	0.099			332 (19.2%)	278 (16.1%)	0.082		
Unknown	11 (0.4%)	10 (0.6%)	− 0.022			5 (0.3%)	9 (0.5%)	− 0.036		
Adjuvant therapy				< 0.001	4)				< 0.001	4)
No radiation or chemotherapy	1360 (44.2%)	836 (46.0%)	− 0.039			792 (45.9%)	789 (45.7%)	0.002		
+ Radiation therapy	37 (1.2%)	44 (2.4%)	− 0.099			24 (1.4%)	39 (2.3%)	− 0.065		
+ Chemotherapy	1353 (43.9%)	687 (37.8%)	0.129			737 (42.7%)	659 (38.2%)	0.094		
+ Radiation and chemotherapy	327 (10.6%)	250 (13.8%)	− 0.094			173 (10.0%)	240 (13.9%)	− 0.118		
Missing	3 (0.1%)	0 (0%)	–			1 (0.1%)	0 (0%)	–		

Statistical comparisons were made using (1) Student's *t* test, (2) the chi-squared test, (3) Cuzick's Wilcoxon-type test for trend, (4) Fisher's exact test

*AIBCES* Authorized Institute for Board Certified Esophageal Surgeons

Comparison of the Kaplan–Meier survival curves using log-rank tests indicated a significant difference between the AIBCES and Non-AIBCES groups at cStages I–IV in the crude data (Table 2). The 5-year survival rate for patients with cStage I cancer treated at an AIBCES vs. Non-AIBCES was 76.0% vs. 69.4%; cStage II, 55.4% vs. 46.4%; cStage III, 41.5% vs. 30.1%; and cStage IV, 31.2% vs. 23.5% (Table 2).

**Table 2:** Five-year survival rates among patients in the AIBCES and Non-AIBCES groups by clinical factor

	Overall	AIBCES	Non-AIBCES	*P*	
Numbers of patients		3080	1817		
Time at risk (days)		4,668,135	2,459,343		
Death		1524 (49.5%)	1042 (57.3%)	< 0.001	1)
5-year survival rate (%, Kaplan–Meier method)					
Age					
< 70	53.7	56.9	48.0	< 0.001	2)
≧ 70	44.5	47.2	40.4	0.004	2)
Sex					
Female	61.7	66.9	52.9	< 0.001	2)
Male	48.9	51.7	44.2	< 0.001	2)
T classification (*n* = 4785)					
T ≤ 2	62.4	65.6	57.3	< 0.001	2)
T ≥ 3	37.5	41.1	30.8	< 0.001	2)
N classification (*n* = 4799)					
N0	62.2	67.0	54.7	< 0.001	2)
N1–3	41.0	43.3	36.7	< 0.001	2)
M classification (*n* = 4812)					
M0	52.9	56.4	47.1	< 0.001	2)
M1	28.9	31.6	22.8	0.002	2)
Stage (UICC, *n* = 4787)					
Stage 0	75.0	81.8	60.0	0.26	2)
Stage I	73.7	76.0	69.4	0.003	2)
Stage II	51.6	55.4	46.4	< 0.001	2)
Stage III	37.6	41.5	30.1	< 0.001	2)
Stage IV	28.8	31.2	23.5	0.004	2)
Adjuvant therapy (*n* = 4894)					
No radiation or chemotherapy	59.9	64.1	53.1	< 0.001	2)
+ Radiation therapy	9.9	10.8	9.1	0.66	2)
+ Chemotherapy	48.8	51.3	44.0	0.004	2)
+ Radiation and chemotherapy	29.2	28.2	30.4	0.97	2)

Statistical comparisons were made using (1) the Chi-squared test, (2) log-rank test

*AIBCES* Authorized Institute for Board Certified Esophageal Surgeons

Multivariable Cox proportional hazard analysis stratified by clinical stage and adjuvant therapy (*n* = 4785) revealed that AIBCES vs. Non-AIBCES is significantly related to better survival (adjusted HR 0.78) (Table 3).

**Table 3:** Multivariable Cox proportional hazard analysis stratified by stage and adjuvant treatment (*n* = 4785)

Factor	HR	*P*	95% CI
Age ≧ 70 vs. < 70	1.37	< 0.001	1.26–1.49
Male vs. Female	1.56	< 0.001	1.38–1.76
AIBCES vs. Non-AIBCES	0.78	< 0.001	0.72–0.84

*AIBCES* Authorized Institute for Board Certified Esophageal Surgeons

**Fig. 1:**
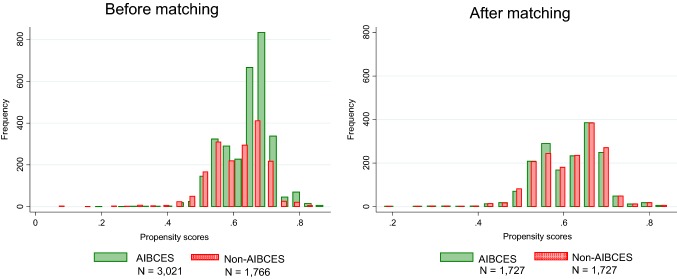
Distribution of propensity scores before and after matching

**Fig. 2:**
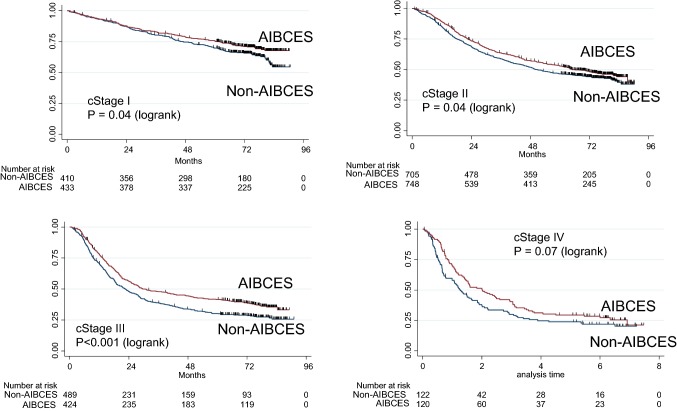
Kaplan–Meier curves for overall survival of patients with cStage I, II, III, or IV thoracic esophageal cancer operated on at an Authorized Institute for Board Certified Esophageal Surgeons (AIBCES) or a Non-AIBCES after propensity score matching. There are significant differences in 5-year overall survival at cStages I–III

Propensity scores were used to match the likelihood of being treated at an AIBCES between the groups. After propensity score matching, the demographic and clinical characteristics before treatment were adequately balanced between the 1727 pairs of the AIBCES and Non-AIBCES groups: standardized difference < 0.100 (Table 1, Fig. 1). As a result, adjuvant chemo- or chemoradio-therapy was administered to 54% of patients treated at an AIBCES and to 52% treated at a Non-AIBCES in this analysis. Comparison of the Kaplan–Meier survival curves using log-rank test indicated a significant difference in the 5-year survival rates between the AIBCES and Non-AIBCES groups for patients with cStage I–III disease (Fig. 2). Cox proportional hazard regression analyses stratified by the type of adjuvant treatment showed that patients receiving treatment at an AIBCES had a significantly lower hazard ratio (HR 0.82, 95% CI 0.75–0.90) than those treated at a Non-AIBCES.

## Discussion

This study found a significantly better 5-year survival rate among cStage I–IV thoracic esophageal cancer patients treated with esophagectomy at an AIBCES as compared to those treated at a Non-AIBCES before and after propensity score matching. Cox proportional hazard regression analyses before and after propensity score matching showed that, in consideration of clinical factors, patients receiving treatment at an AIBCES had a significantly lower hazard ratio than those treated at a Non-AIBCES.

There are several variables that are indicative of the quality and accuracy of surgery for thoracic esophageal cancer. The surgery-related death rate and rate of surgical complications are often used to assess short-term surgical outcomes. In addition, the overall survival rate is a particularly valuable indicator of the quality of surgery for thoracic esophageal cancer because it reflects the thoroughness and/or extent of lymph node dissection, and it is directly influenced by surgical failures, including operation-death and surgery-related death caused by surgical complications. On the other hand, overall survival data require long-term and accurate data management. On this point, at the National Database of Hospital-based Cancer Registries, the precise registry rules and definitions are covered by the tumor registrar training programs, and the data quality is ensured through consistency-checking software provided by the National Cancer Center [2]. Moreover, survival information was often provided through referral of the patient’s residency card, which makes this information extremely reliable.

Clinical stage registration is essential for esophageal cancer, as neoadjuvant therapy is usually administered for advanced cancers. When discussing the quality and accuracy of esophagectomy for thoracic esophageal cancer, cStage must be taken into consideration. For cStage I thoracic esophageal cancer, the standard treatment strategy is surgery alone, which consists of esophagectomy, extensive lymph node dissection from the neck to the abdomen, and reconstruction [5, 6]. The new comprehensive registry of esophageal cancer in Japan (2012) revealed that 903 cStage I patients received esophagectomy, whereas 118 cStrage I patients received definitive chemoradiotherapy [7]. This indicates that a large majority of patients with cStage I esophageal cancer underwent surgery as a definitive treatment in 2012, and few of those patients received adjuvant therapy. This suggests the difference in 5-year survival between AIBCES and Non-AIBCES for cStage I patients directly reflects the surgical quality and accuracy of the lymph node dissection. Our previous report failed to show a statistically significant survival advantage of treatment at an AIBCES for cStage I patients [1]. In the present study, however, we analyzed the data with more than twice as many patients and added the propensity score matching method, which enabled us to clearly show a survival advantage to receiving esophagectomy at an AIBCES.

At cStages II–III, a standard treatment in Japan is neoadjuvant chemotherapy (chemoradiotherapy) plus esophagectomy [5, 6]. When treating patients with cStage II–III disease, it is necessary to have comprehensive knowledge and excellent surgical technique. Our earlier report demonstrated the survival advantage of treatment at an AIBCES in cases of cStage II–III esophageal cancer [1]. However, there was a difference in the background between the two groups, especially in the proportion of patients receiving adjuvant therapy, which was reflected by the survival outcome. We resolved this problem by stratifying adjuvant therapies in the univariate and multivariable analyses, which revealed that the institute certification is a significant independent factor for 5-year survival. Tsukada et al. reported that patients at center hospitals were more likely to receive neoadjuvant therapy for esophageal cancer [8]. In the present study, therefore, we used propensity score matching to ensure that the proportions of patients receiving adjuvant therapy were nearly equal in AIBCESs and Non-AIBCESs (54.1% and 54.3%). Nevertheless, there was significant difference in 5-year survival between the two groups. Our results thus clearly provide promising evidence indicating an advantage to receiving esophagectomy at an AIBCES for cStage II–III patients.

For patients with UICC cStage IV esophageal cancer, surgery was performed in selected patients with resectable N4 lymph nodes, such as the supraclavicular lymph nodes or those along the celiac artery. In Japan, however, supraclavicular lymph nodes are usually considered to be regional nodes and are dissected as standard surgical procedure. Consequently, comparison of the survival difference between the two cStage IV groups is valuable, as it would provide an avenue for discussion of the impact surgical quality and accuracy in more advanced esophageal cancers and of the potential for surgical control in such cases. Unfortunately, however, we cannot use the Japanese cStage IV classification in this study [9, 10]. We anticipate that a future study using the Japanese classification will reveal a further advantage to receiving esophagectomy at an AIBCES.

As mentioned, in the original data, the clinical backgrounds of the study populations differed between the patients treated at an AIBCES or Non-AIBCES, and we employed propensity score matching analysis to compare the survival rates more objectively. Propensity score matching is an effective method to control for confounding in observational studies. In the present study, we analyzed real-world data, making it necessary to avoid possible selection bias. Propensity score matching must, therefore, incorporate as many confounders as possible, which can greatly reduce the number of samples. For this reason, the number of pairs that match in the propensity score often becomes small, and the validity of the study falls. This can make it questionable whether the obtained results can be generalized to the whole population being examined. Fortunately, in the present study, the number of patients was large enough that this problem did not arise [11].

The strengths of our study include the following. Through the use of a large sample and propensity score matching, we were able to minimize potential biases while maintaining a higher degree of power. Another important merit of the study is the accuracy of the survival data achieved through the use of big data. Moreover, in Japan, the treatment strategy is uniformed throughout the country. As a result, the conclusions drawn from this study are based on high-quality and reliable data. On the other hand, our study design has several limitations. First, the cancer data were collected only from Designated Cancer Care Hospitals and only for first-course treatments provided by the registering facility. As a result, the data on who received esophagectomy were incomplete. For example, salvage esophagectomy for a recurrent tumor after definitive chemoradiotherapy is a complex procedure that has a higher frequency of severe complications and surgery-related death [12]. Although there is an even greater need for surgical quality and accuracy in salvage surgery, a complete dataset on who received salvage esophagectomy is not available. Second, our data did not include the presence of comorbidity and performance status because the registry does not have the information. Third, there was also a question as to whether the difference in survival between AIBCESs and Non-AIBCESs reflects the certification itself or the treatment volumes. Naturally, certified hospitals tend to have a larger volume of patients than non-certified hospitals. Therefore, we were unable not separate the effect of these two factors in this study.

In conclusion, our findings indicate that undergoing esophagectomy at an AIBCES certified by the JES provides an evident advantage for long-term patient survival. The institute certification system by JES will contribute to the future establishment of a more appropriate surgery delivery system for thoracic esophageal cancer.
